# Complete genome sequences and comparative secretomic analysis for the industrially cultivated edible mushroom *Lyophyllum decastes* reveals insights on evolution and lignocellulose degradation potential

**DOI:** 10.3389/fmicb.2023.1137162

**Published:** 2023-03-23

**Authors:** Lili Xu, Wujie Yang, Tianmei Qiu, Xia Gao, Hongyong Zhang, Shuliang Zhang, Hui Cui, Lizhong Guo, Hailong Yu, Hao Yu

**Affiliations:** ^1^Shandong Provincial Key Laboratory of Applied Mycology, School of Life Sciences, Qingdao Agricultural University, Qingdao, Shandong, China; ^2^National Engineering Research Center of Edible Fungi, Institute of Edible Fungi, Shanghai Academy of Agricultural Sciences, Shanghai, China; ^3^Shandong Agricultural Technology Extending Station, Jinan, Shandong, China; ^4^Dezhou Academy of Agricultural Sciences, Dezhou, Shandong, China

**Keywords:** edible mushroom, *Lyophyllum decastes*, genome, proteome, secretome, CAZymes

## Abstract

*Lyophyllum decastes*, also known as Luronggu in China, is a culinary edible and medicinal mushroom that was widely cultivated in China in recent years. In the present study, the complete high-quality genome of two mating compatible *L. decastes* strain was sequenced. The *L. decastes* LRG-d1-1 genome consists of 47.7 Mb in 15 contigs with a contig N90 of 2.08 Mb and 14,499 predicted gene models. Phylogenetic analysis revealed that *L. decastes* exhibits a close evolutionary relationship to the *Termitomyces* and *Hypsizygus* genus and was diverged from *H. marmoreus*  ~ 45.53 Mya ago. Mating *A* loci of *L. decastes* compose of five and four *HD* genes in two monokaryotic strains, respectively. Mating *B* loci compose of five *STE* genes in both two monokaryotic strains. To accelerate the cross-breeding process, we designed four pairs of specific primers and successfully detected both mating types in *L. decastes*. As a wood-rotting mushroom, a total of 541 genes accounting for 577 CAZymes were identified in the genome of *L. decastes*. Proteomic analysis revealed that 1,071 proteins including 182 CAZymes and 258 secreted enzymes were identified from four groups (PDB, PDB + bran, PDB + cotton hull, and PDB + sawdust). Two laccases and a quinone reductase were strongly overproduced in lignin-rich cultures, and the laccases were among the top-3 secreted proteins, suggesting an important role in the synergistic decomposition of lignin. These results revealed the robustness of the lignocellulose degradation capacity of *L. decastes*. This is the first study to provide insights into the evolution and lignocellulose degradation of *L. decastes*.

## Introduction

*Lyophyllum decastes* is a delicious edible and medicinal mushroom that was firstly industrially cultivated in Japan and widely cultivated in China and other Asia country in recent years (Masahito, 1991; Kinuta et al., 1996; Wei et al., 2006). The shape of *L. decastes* fruiting body looks like an antler therefore it was called Luronggu in China. China is the largest Luronggu producer in the world, and the yield of *L. decastes* in China is about 11,590 tone in 2020. The production areas are mainly distributed in Shandong, Jiangxi, Shanghai, and Hebei provinces. Shandong is the largest production area in China, which produce 7,380 tons (63.7%) in 2020 and 21,805 tons in 2021. *Lyophyllum decastes* uses lignocellulose-rich media, such as sawdust, bran, and corncob, for fruiting body production. Because of its culinary taste and good processing characteristics, *L. decastes* is favored by consumers and chefs.

*Lyophyllum decastes* is proved to be a health-promoting supplement due to its antitumor (Kim et al., 1984; Ukawa et al., 2000; Ding et al., 2022), antidiabetic (Miura et al., 2002), antihypertension (Kokean et al., 2005), anti-hyperlipidemic (Ukawa et al., 2002; Wang T. et al., 2022), immunoregulation (Ding et al., 2022), hepatic-protection (Zhang et al., 2022), radioprotection (Nakamura et al., 2007), and skin lesion protection effects (Ukawa et al., 2007). Polysaccharides were the major bioactive compounds characterized in *L. decastes* (Ukawa et al., 2000; Ding et al., 2022; Wang T. et al., 2022; Zhang et al., 2022). Pokhrel et al. investigated the optimistic submerged culture conditions for mycelial yield and polysaccharide production by *L. decastes* and found that a concentration of glucose 3% + yeast extract 1% was a suitable condition for submerged culture (Pokhrel and Ohga, 2007). Two polysaccharides’ structures have been resolved to date. Uwaka et al. purified polysaccharides from a hot-water extract of fruiting bodies of *L. decastes* by ion exchange and gel permeation chromatography. The structures of the purified polysaccharides were (1 → 3)-*β*-D-glucan, (1 → 6)-*β*-D-glucan, and (1 → 3, 1 → 6)-*β*-D-glucan, respectively, (Ukawa et al., 2000). It has proved that the *β*-glucans have *β*-(1 → 3) linkages in the main chain of the glucan and additional *β*-(1 → 6) branch points that are needed for their antitumor actions (Wasser, 2002; Ren et al., 2012). This partially explains the antitumor activities of water extract of *L. decastes*. Ding et al. purified another polysaccharide from the fruiting body of *L. decastes* using the DEAE-52 ion-exchange column. Structural analysis revealed four (1 → 4)-*ɑ*-D-Glcp moieties with branches at the 6-O position and the branches consisted of two (1 → 6)-*β*-D-Galp moieties (Ding et al., 2022). In addition to polysaccharides, an uncommon amino acid, 6-hydroxy-L-tryptophan was isolated from the hot water extract of the lyophilized *L. decastes* fruiting body and showed an inhibition effect on the activity of tyrosinase from *Agaricus bisporus*, which may be used as a potential medicine (Ishihara et al., 2019). The results proved that *L. decastes* is a good-tasting nutritional and functional food.

Although the production yield of *L. decastes* keeps increasing in China, the research on this mushroom is mainly focused on the isolation and characterization of bioactive constituents. Little information has been reported related to the molecular mechanism of bioactive compound synthesis, physiological phenomena, or substrates utilization. Goldstein et al. isolated a *ɑ*-galactosyl-binding lectin from the fruiting bodies of *L. decastes* (Goldstein et al., 2007). The structure of the lectin was further resolved by Eerde et al. (2015). Without genomic information, the lectin amino acid sequence was determined by cloning from a cDNA library using partial sequences determined by Edman sequencing and MS analysis (Goldstein et al., 2007). Hu et al. reported that ultrasonic treatment of *L. decastes* fruiting body could reduce the browning index by 21% retaining a higher level of phenols, flavonoids, and 9 kinds of amino acids. Higher enzyme activities of antioxidant enzymes, such as catalase, peroxidase, cytochrome *c* oxidase, and higher ATP concentration may be ascribed to the increased shelf life of this mushroom (Hu et al., 2022). Wang et al. fabricated a chitosan/zein/tea polyphenol film which was used in the postharvest storage of *L. decastes*. The film showed the effectiveness of maintaining the color, texture as well as the integrity of cell membrane. The low enzyme activity of polyphenol oxidase and peroxidase and the higher activity of antioxidant enzymes may be related to the quality improvement of this mushroom (Wang X. et al., 2022). However, the molecular mechanism underlining the biochemical evidence was still unknown. Sunagawa et al. firstly reported the transformation of *L. decastes* (Sunagawa et al., 2007). The pHHM192 plasmid containing the hygromycin B reporter gene was transformed into *L. decastes* N1 strain by particle bombardment. However, no gene was edited in this experiment. The main limitation form these researches is the lack of genomic information on *L. decastes*.

Genomic information is important for the process of genetic engineering and precision breeding (Ohm et al., 2010; Morin et al., 2012; Bao et al., 2013; Chen et al., 2016; Liang et al., 2018; Yuan et al., 2019). With the development of genome sequencing technologies, genomes of a lot of commercially cultivated mushrooms have been sequenced (Liu et al., 2018; Yuan et al., 2019; Gong et al., 2020; Li et al., 2022; Yu et al., 2022). Genomes could be applied to the study of evolutionary processes in different mushroom species (Wu et al., 2018; Zhang et al., 2021; Wu et al., 2022). Identification of molecular markers, such as sex-linked markers and SSR markers, based on the genome could help us to identify the mushroom cultivar and speed up mushroom breeding (Fang et al., 2020; Gong et al., 2020; Yu et al., 2022). CAZymes play key roles in lignocellulose degradation and geochemical circulation (Garron and Henrissat, 2019; Pallister et al., 2020). White-rot fungi, including most of edible mushrooms, are important repository of CAZymes. Therefore, genome sequencing of edible mushroom will further our understanding of CAZymes, which also play important roles in environmental adaptation, bioactive compounds synthesis, biofuel production, and substrate utilization by mushrooms (Morin et al., 2012; Dai et al., 2019; Park et al., 2019; Li et al., 2022; Yu et al., 2022). With genomic information, more genes related to important physiological characteristics and key agricultural traits could be identified (Wang et al., 2018, 2021a). Besides, the genome could promote the proteome and transcriptome research of mushrooms (Lin et al., 2022).

The mitochondrial genome of *L. decastes* was sequenced recently (Li et al., 2019), and one genome of *L. decastes* has been deposited in the GenBank. However, high quality genome sequence of *L. decastes* was not reported to date. In the present study, a high-quality genomes for *L. decastes* were reported to decipher the genome organization of *L. decastes* and facilitate development of genetic and genomic tools essential for *L. decastes* breeding. The genome assembly of *L. decastes* and proteomic analysis will facilitate the better utilization of this mushroom in multiple aspects.

## Materials and methods

### Growth conditions

**Figure 1:**
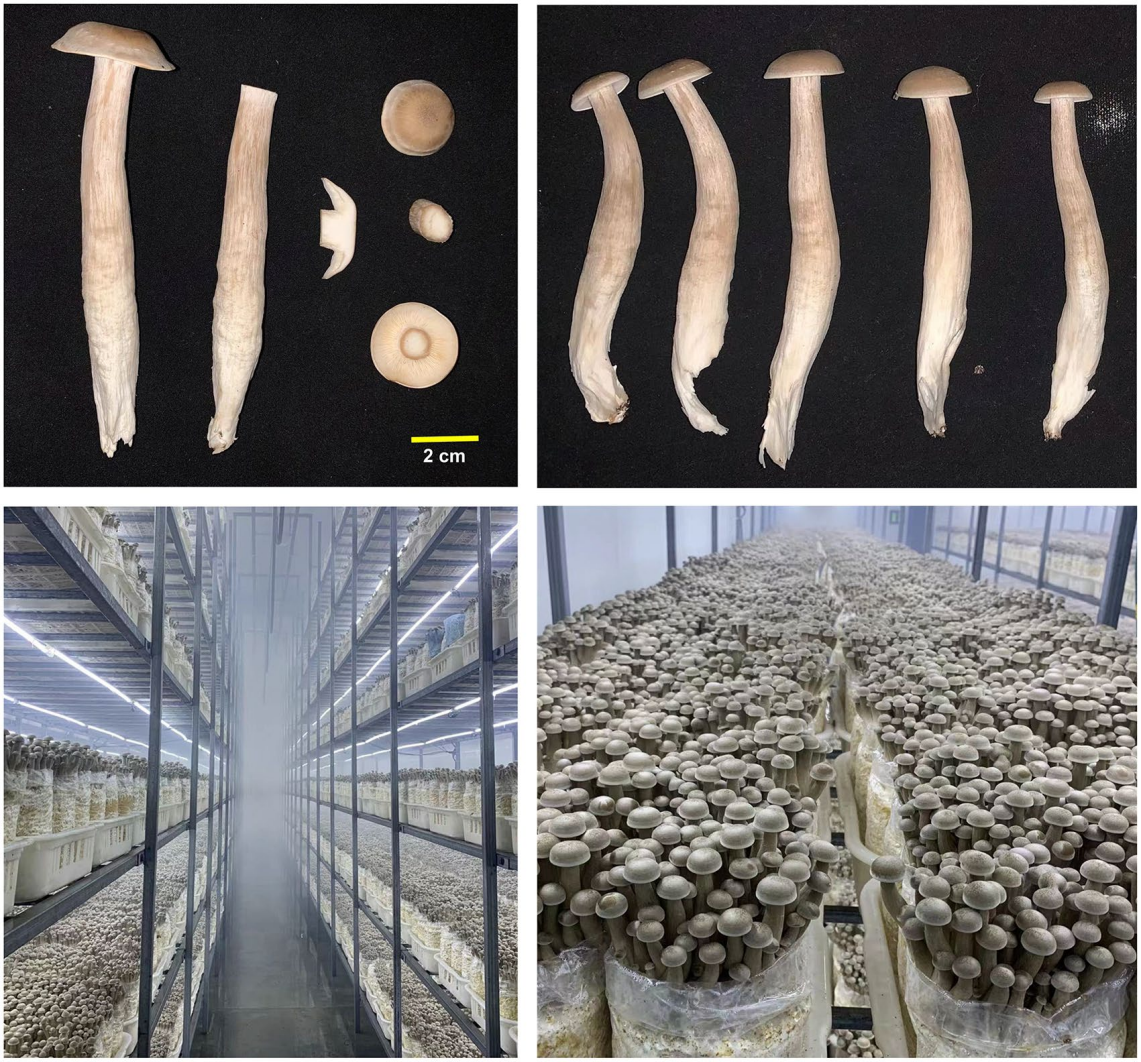
Industrially cultivated *Lyophyllum decastes* fruiting body.

For industrial production, the temperature for vegetable growth of *L. decastes* is ~23°C. After ~60 days of cultivation, the surface mycelia were scratched and the temperature was adjusted to 16–18°C, 90%–95% humidity. The fruiting bodies were harvested 25–30 days after scratching. The dikaryotic strain LRG-d1 was isolated from the fruiting body of industrially cultivated *L. decastes* (Figure 1). The Basidiomycetes spores were collected from the fruiting body of strain LRG-d1. The spores were spread on the PDA plates by serial dilution. The monokaryotic strain without clamp connection was isolated and verified by micro-examination. The mating compatibility between different monokaryotic strains was determined by confrontation culture. Monokaryotic strains that could form clamp connections were selected for further analysis as previously described (Li et al., 2022). Three monokaryotic strains were cultivated on a PDA plate covered with cellophane as previously described (Li et al., 2022). After spreading all the plate, the mycelia were scraped from the cellophane, frozen in liquid nitrogen, and stored at −80°C for genome sequencing and RNA-Seq.

### WGS and genome assembly

Genome DNA was extracted from mycelia of monokaryotic strain LRG-1 using the NucleoBond HMW DNA kit (Macherey-Nagel, Düren, Germany) according to the manufacturer’s instructions. The Nanopore library was constructed using Oxford Nanopore LSK-109 kit, and the Nanopore library was sequenced on the PromethION platform by Benagen Technology Co., Ltd. (China, Wuhan). After quality filtered using Filtlong v0.2.0 (Wick, 2017), a total of 6.55 Gb and 9.19 Gb clean raw data were generated for strain LRG-d1-1 and LRG-d1-5, respectively. Illumina paired-end sequencing was sequenced on an Illumina NovaSeq 6000 platform under 150 bp mode in the same company. The raw fastq data were filtered using fastp software, and 4.86 Gb, 3.51 Gb, and 5.27 Gb raw data were generated after filtration for strain LRG-d1-1, LRG-d1-5, and LRG-d1-2, respectively.

Raw Oxford Nanopore reads were assembled using flye v2.8.3-b1695 and NextDenovo v2.4.0 software with default parameters. The data reads were also assembled using necat with parameters: GENOME_SIZE = 48 m and CNS_OUTPUT_COVERAGE = 70. The assembled contig was polished through two iterations using Racon^1^ with Nanopore reads and default parameters. The polished contigs were further polished with Pilon v1.24 through two iterations using filtered Illumina reads (Walker et al., 2014). GC content = (G + C)/(A + T + G + C).

### Gene prediction and functional annotation

Genes were predicted with Augustus software using laccaria_bicolor models (Stanke et al., 2006). Genes were predicted using GeneMark-ES v 4.69 for self-training prediction using default parameters (Ter-Hovhannisyan et al., 2008). RNA-Seq data were mapped using stringtie software (Pertea et al., 2015) and the results were used for model training with Augustus software, the genes were also predicted using the trained model based on RNA-Seq transcripts. The three prediction genes were integrated using EVidenceModeler software (Haas et al., 2008). Functional annotations of predicted protein-coding sequences (CDSs) against EggNGOmapper, Pfam, and SwissProt were performed as previous description (Yu et al., 2022). CAZymes of the *L. decastes* genome were annotated using dbcan version v3.0.2 software with Hmmer search engine and default parameters as previously described (Yu et al., 2022). The signal peptide of a protein was predicted using SignalP v5.0b software (Almagro et al., 2019). The trans-membrane structure was predicted using TMHMM v2.0c (Chen et al., 2003). Protein that has signal peptide without trans-membrane structure was secreted protein.

### Comparative genomic analysis

The pairwise average nucleotide identity (ANI) analysis was performed using fastANI software (Jain et al., 2018). Collinearity analysis was performed using MCScanX software (Wang et al., 2012). Orthogroups analysis was using OrthoFinder software (Emms and Kelly, 2019). Divergent time analysis and gene family evolution were performed using Bayesian software packages MCMCTree and CAFE v5.0 software, respectively, (Yang, 2014; Mendes et al., 2020).

### Identification of the mating locus

The mating-type loci of *L. decastes* were analyzed using sequence alignment with homeodomain (HD) genes and pheromone receptor genes (STE) genes from *Hypsizygus marmoreus* and *Pleurotus giganteus* as the query sequences (Wang et al., 2021b; Yu et al., 2022). The functions of the identified genes were further confirmed using Blastp tools in the NCBI database. Gene cluster structure was visualized using integrative genomics viewer software (Thorvaldsdóttir et al., 2013).

### Submerged cultivation of *Lyophyllum decastes* mycelia and secreted protein extraction

Dikaryotic strain LRG-d1 was inoculated and cultivated on a PDA plate. Three mycelial plugs (8 mm diameter) were cut from the margin of seed agar cultures and inoculated into 50 mL PDB media (extract from 200 g potato, 20 g dextrose) and 50 ml lignin-rich media (50 mL PDB media add 1 g sawdust, 1 g cottonseed hull, and 1 g bran, respectively) in 250 ml triangular flasks, respectively. Submerged cultivation of *L. decastes* mycelia was performed at 25°C in darkness at 150 rpm. Three replicates were performed for each condition.

### Proteomic analysis and data processing

After 10 days of cultivation, the culture was filtered using 6-layer absorbent cotton gauze. The filters were centrifuged at 10,000 rpm for 20 min. The secreted proteins in the supernatant were extracted using phenol extraction coupled with ammonium acetate precipitation as described by Isaacson et al. (2006). Protein pellets were re-dissolved in 4% SDS buffer (in 100 mM Tris–HCl buffer, pH 7.6) and digested using trypsin with STrap methods as described by Zougman et al. (2014). LC-MS analysis was performed in DDA mode as in our previous description without changing (Lin et al., 2022), and the chromatographic time is 90 min. Three biological replicates were performed for each group.

Proteins were identified with at least two unique peptides with a false discovery rate lower than 0.05 using MaxQuant software with default parameters (Tyanova et al., 2016a). For protein expression quantification, MaxQuant software was used to calculate the intensity-based absolute quantification (iBAQ) abundances for each protein. Abundance data obtained from MaxQuant software was processed using Perseus software (Tyanova et al., 2016b). Proteins were normalized using sum normalization methods. Visualization was performed using the R program with ggplot2 and heatmap2 packages.

## Results and discussion

### Genome assembly and annotation

Monokaryotic strain LRG-d1-1, LRG-d1-2, and LRG-d1-5 were isolated from the basidiospore of dikaryotic strain LRG-d1 (Figure 1). Monokaryotic *L. decastes* strain LRG-d1-2 and LRG-d1-5 were mating compatible strains to strain LRG-d1-1. To comprehensive analyze the genome of *L. decastes*, the genomes of three monokaryotic strains were sequences.

**Figure 2:**
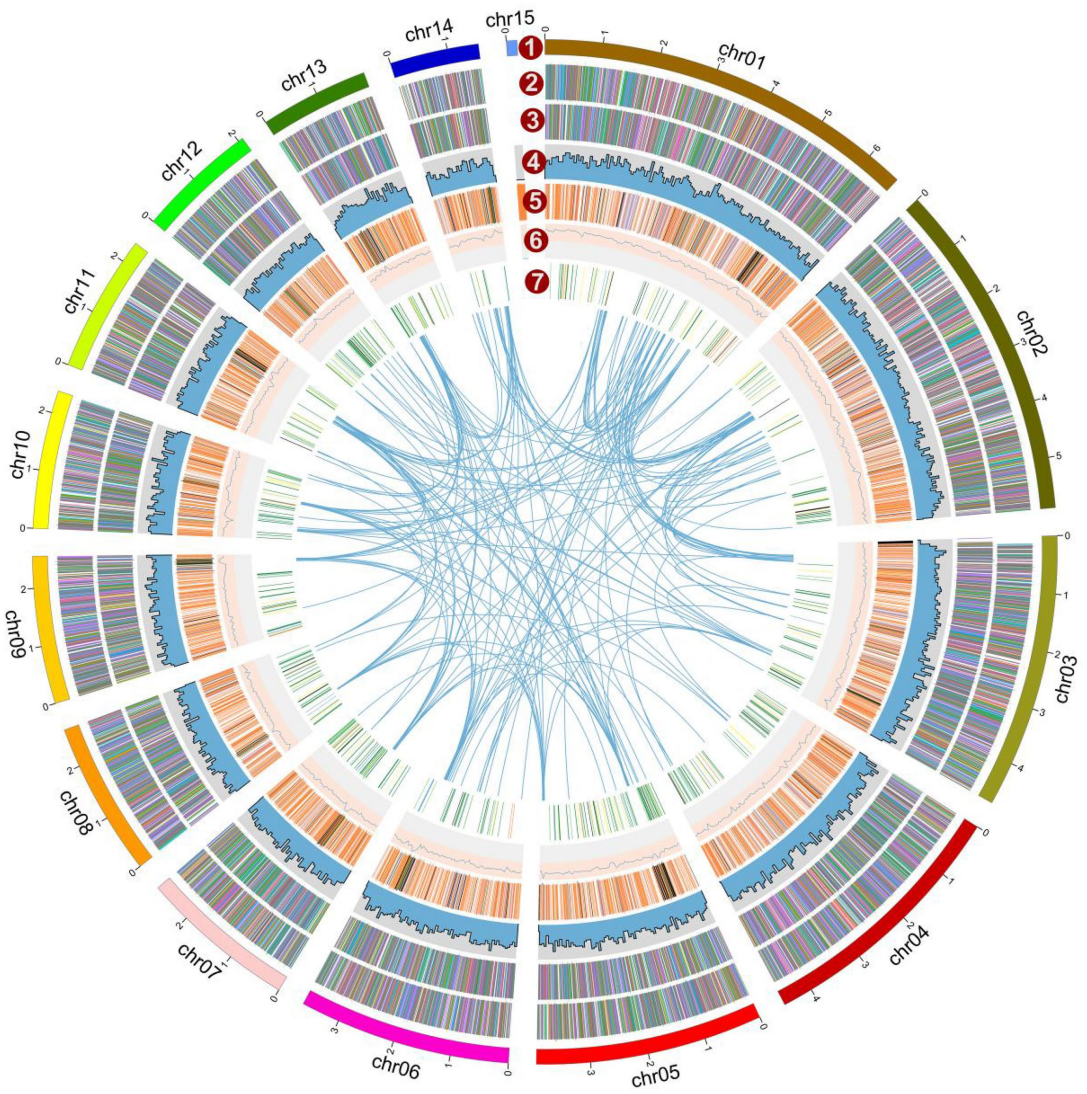
Overview of the *Lyophyllum decastes* genome assembly and gene prediction. The outermost layer 1 of colored blocks is a circular representation of the 15 contigs, with a scale mark labeling each 1 Mb. Layer 2 and 3, predicted genes in forward and reverse strand of genome; Layer 4, gene density; Layer 5, repeat sequences; Layer 6, GC content; Layer 7, CAZymes coding genes. Links within and between chromosomes represent collinear blocks generated from MCScanX. The window size is 50 kb. The plot was visualized using Circos software.

The genome of the *L. decastes* monokaryotic strain LRG-d1-1 strain was sequenced using Oxford Nanopore and Illumina sequencing platforms. *De novo* assembly was performed with Necat software using the long reads generated by the PromethION platform of Oxford Nanopore Technologies. The final genome assembly of strain LRG-d1-1 consists of 15 contigs, which spans 47.72 Mb in total, with the contig N50 size of 3.92 Mb and N90 size of 2.08 Mb (Table 1; Figure 2). The average GC content of the CDSs of *L. decastes* LRG-d1-1 (50.25%) is similar to that of *L. decastes* JCM 30590 (50.00%), *Lyophyllum semitale* D66 (50.06%) and *Lyophyllum shimeji* JCM 30591 (50.38%), while lower than that of *L. atratum* CBS 144462 (51.34%). Repeat annotation revealed that 25.67% (12.25 Mb) of the assembled *L. decastes* genome comprised repeat elements (Supplementary Table 1). We compared the proportion of repeat elements in seven edible mushrooms, *Agaricus bisporus* var. burnettii H119_p4 (15.31% in 30.7 Mb genome), *Flammulina velutipes* 6–3 (12.79% in 38.1 Mb genome), *Lentinula edodes* L808 (27.23% in 45.9 Mb genome), *Pleurotus ostreatus* PC15 (9.08% in 34.3 Mb genome), *Pleurotus giganteus* zhudugu2 (11.36% in 40.0 Mb genome), *Stropharia rugosoannulata* A15 (17.80% in 47.9 Mb genome), and *L. decastes* LRG-d1-1. It seems that larger genome has larger repeat elements proportion. Similar to most edible mushrooms, LTR elements were the dominant class of repeat elements. Gypsy/DIRS1 LTR elements accounted for 10.67% of the genome. The genome of *L. decastes* strain LRG-d1-2 was sequenced using the Illumina platform and the genome of strain LRG-d1-5 was sequenced using Illumina and Nanopore platforms. The final genome assembly of strain LRG-d1-5 consists of 18 contigs, which spans 42.98 Mb in total, with the contig N50 size of 3.53 Mb. The genome size of strain LRG-d1-2 is 40.04 Mb in 2,400 contigs.

**Table 1:** *De novo* genome assembly and features of *Lyophyllum decastes*.

Characteristics	LRG-d1-1	LRG-d1-5	LRG-d1-2
Genome assembly size	47.72 Mb	42.98 Mb	40.04 Mb
Contigs	15	18	2,400
Longest scaffold	6.62 Mb	5.16 Mb	16,686 Kb
Contig N50	3.92 Mb	3.53 Mb	90.6 Kb
Contig N90	2.08 Mb	1.28 Mb	9,774 bp
GC	50.25%	50.38%	50.35%
Sequencing platform	Oxford Nanopore, Illumina	Oxford Nanopore, Illumina	Illumina

The length of the contigs is larger than 500 bp.

Based on a comprehensive strategy of *ab initio* gene prediction and homology evidence from RNA-Seq transcripts, 14,499 non-redundant gene models were predicted from the genome of *L. decastes* with an average sequence length of 1,652.2 bp and an average exon number of 6.25 (Table 2; Figure 1). The total length of CDSs was 23.95 Mbp accounting for 50.2% of the total genome. The predicted gene sequence was annotated functionally by sequence alignment against sequences in EggNOGmapper, SwissProt, Pfam, and CAZymes databases. Among the annotated genes, 11,669 and 6,415 genes were classified by the EggNOGmapper database and SwissProt database (Supplementary Table 3), respectively. A total of 8,655 (59.7%) genes were annotated by the Pfam database based on the similarity of protein domains. The completeness of gene prediction was evaluated with 758 BUSCO genes from fungi_odb10, of which 725 BUSCOs (95.7%) were complete (Supplementary Figure 1). Which indicated a high quality of the genome assembly and high fidelity of gene prediction.

**Table 2:** Characteristics of the gene prediction of *Lyophyllum decastes* (LRG-d1-1).

Content	Number/length
Gene number	14,499
Total concatenated gene length	23,955,415 bp
Average gene length	1,652.21 bp
Average exon length	264.52 bp
Average exon number	6.25
Average intron length	74.00 bp

### Comparative genomic analysis

The average nucleotide identity (ANI) was analyzed to estimate genomic differences and relatedness between sequenced *Lyophyllum* strains. As shown in Supplementary Figure 2, four *L. decastes* strains showed high genomic similarities width ANI value higher than 92%. The results confirmed that these strains are from the same species. The ANI values between different species were lower than 83. The *Lyophyllum* strains showed no ANI value with strains from the *Trichoderma* genus (data not shown). The results confirmed the phylogenetic relationship of these strains based on morphological features and ribosomal sequences.

**Figure 3:**
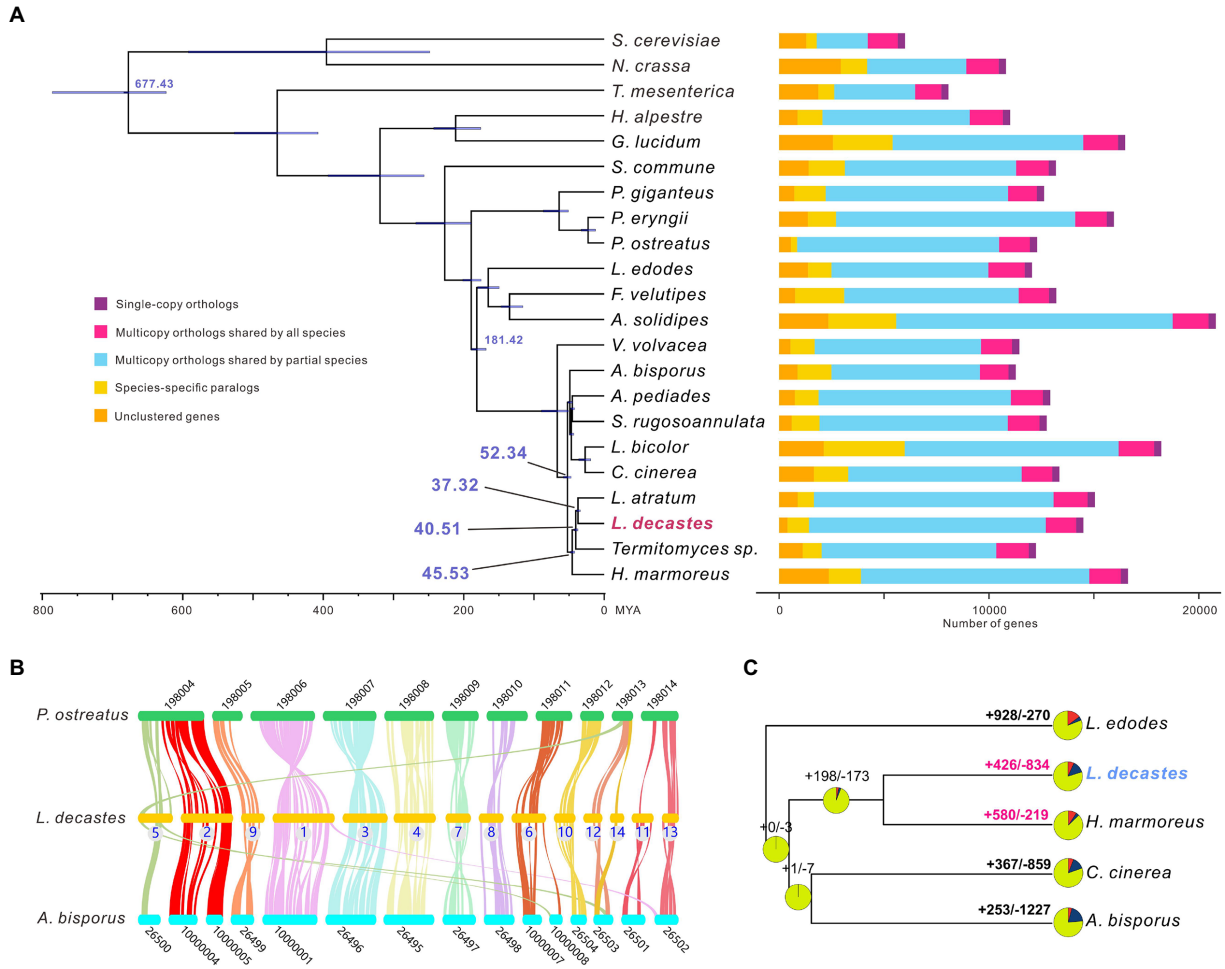
Evolutionary and comparative genomic analysis of *Lyophyllum decastes*. **(A)** Fungi phylogenetic tree. Phylogenetic tree inferred from 346 single-copy orthologs among 22 fungi species identified using OrthoFinder. Divergence timings were indicated using transparent blue bars at the internodes with 95% highest posterior density. Single-copy orthologs are defined as orthologs that were present as a single-copy gene in all 22 species. Multicopy orthologs shared by all species represent the gene groups present in all species with a gene number of >1 in at least one species. Multicopy orthologs shared by partial species represent gene groups present in more than one and less than 22 species with a gene number of >1 in at least one species. Species-specific paralogs represent genes uniquely present in only one species. Other genes are classified as unclustered genes. **(B)** The genome collinearity among *L. decastes*, *P. ostreatus*, and *A. bisporus*. Each line connects a pair of collinearity blocks between two genomes. **(C)** Gene family expansion and contraction analysis between five fungi including *L. decastes*.

All-against-all comparisons were performed using OrthoFinder to identify orthologs of *L. decastes* with orthologues in 21 fungi species. Concatenated amino acids from 346 single-copy orthologs were used for phylogenetic reconstruction and species divergence time estimation. Four species from the *Lyophyllaceae* family (*H. marmores*, *L. atratum*, *L. decastes,* and *Termitomyces* sp.) were clustered in one clade (Figure 3A), and the results confirmed that taxonomy based on morphology and ITS sequences. We estimated that the divergence of species from the *Lyophyllaceae* family lineage and *Agaricaceae* family lineage (*A. bisporus*) was estimated to occur ~52.34 MYA (Figure 3A). *Lyophyllum decastes* split from *Termitomyces* sp. around 40.51MYA. *Lyophyllum decastes* and *L. atratum* shared a common ancestor about ~37.32 MYA ago.

For inter-species comparative genomic studies, the synteny of the *L. decastes* genome and two chromosome-scale assembled genome in *Agaricales* (*A. bisporus* and *P. ostreatus*) were analyzed (Figure 3B). Significant collinearity was detected between the three species. Ten contigs in *L. decastes* (chr01, chr02, chr03, chr04, chr06, chr07, chr08, chr10, chr11, and chr13) show collinearity with only one chromosome in *P. ostreatus* and intra-chromosome rearrangements were observed between these chromosomes. Some inter-chromosome rearrangements can also be observed. Three blocks from chr05, chr12, and chr14 show collinearity with chromosome KL198013 in *P. ostreatus*. Similar collinearity was observed between *L. decastes* and *A. bisporus*, only three contigs (chr01, chr05, and chr06) show collinearity blocks with two chromosomes in *A. bisporus*. The results indicated that genome structure variations in *L. decastes* mainly occurred inside a chromosome instead of rearrangement between different chromosomes.

Compared with the ancestor of *L. decastes* and *H. marmoreus*, gene family expansion and contraction analysis classified 426 and 834 gene families as expanded and contracted, respectively (Figure 3C). Among these, 58 were identified as rapidly/significantly evolving families (*p-*value < 0.1), including 39 expanded and 19 contracted (Supplementary Table 4). Functional annotation of the 1,194 expanded gene families revealed that they were mainly involved in transposon (10 gene families) and regulation (including kinases, proteins with SNF2-related domain, and Ankyrin repeats). Therefore, the results indicated that regulation might play important role in the physiological differences between *H. marmoreus* and *L. decastes*. Intragenome comparisons within the *L. decastes* genome revealed that no clear whole-genome duplication (WGD) events during evolution (data not shown), which indicated tandem duplication instead of WGD events might play an important role in the expansion of gene families.

### Identification of carbohydrate active enzymes

**Figure 4:**
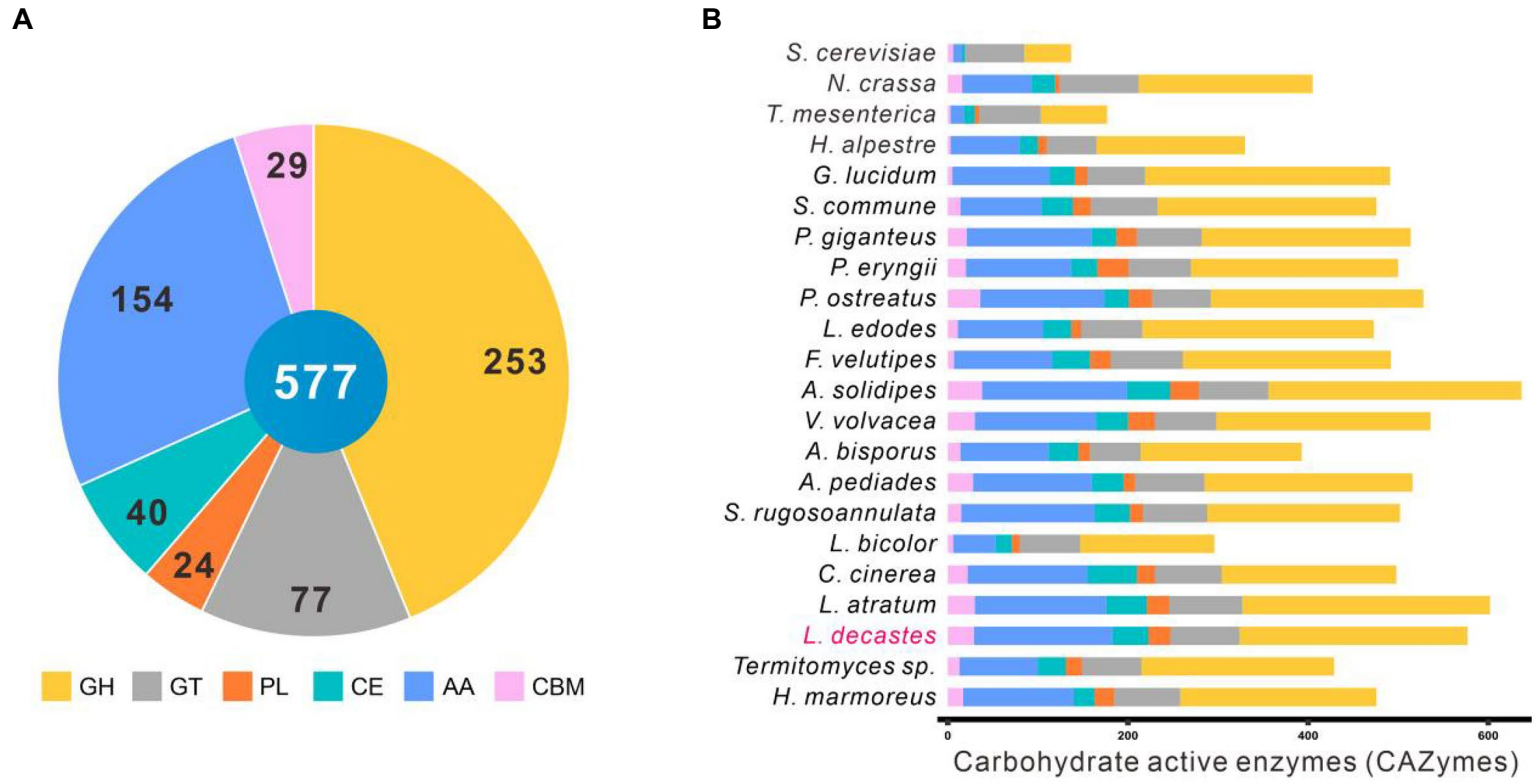
CAZymes in *Lyophyllum decastes* and other 21 fungi. **(A)** The distribution of CAZymes families in *L. decastes*. **(B)** The distribution of CAZymes in all 22 fungi (the same as those in Figure 3).

Complex carbohydrates of plants are the main substrates for white-rot fungi. Carbohydrate-active enzymes (CAZymes) play a critical role in complex carbohydrates metabolism as well as many other important physiological processes, such as development and stress response (Xie et al., 2018; Huang et al., 2020). The CAZymes of the other 22 fungal species were analyzed against the dbCAN2 database with the same parameters. A total of 541 genes accounting for 577 CAZymes terms were identified in the *L. decastes* genome, including 253 GHs, 77 GTs, 24 PLs, 40 CEs, 154 AAs, and 29 CBMs (Figure 4A; Supplementary Table 5).

*Lyophyllum decastes* was a wood-rooting white-rot fungus. The media for *L. decastes* production conpose mainly lignin-rich media. It suggested that L. decastes has powerful lignin modification potential. AA families in the CAZymes database are mainly involved in depolymerization of non-carbohydrate structural components, lignin. Proteins in the AA families were mainly distributed in AA3, AA9, AA1, AA7, AA5, and AA2 categories. Laccases (AA1 family) and class II peroxidases (AA2 family) are important for the degradation of lignin in lignocellulose. A total of 25 laccases in the AA1 category (23 AA1_1, 1 AA1, and 1 AA1_2 category) and 7 class II peroxidases were annotated in the AA2 category in *L. decastes* genome (Supplementary Table 5). The number of laccases is the highest among all the analyzed fungi, which indicated that lignin degradation by *L. decastes* depends mainly on the addition of AA1 subfamilies. The results confirmed the lignin degradation potential of this mushroom, which is consistent with the wood-rich media used in *L. decastes* production.

Interestingly, *Termitomyces* species showed a close evolutionary relationship with *L. decastes*. CAZymes analysis revealed that only 87 AAs (56.5% of those annotated from *L. decastes*) were identified in *Termitomyces* sp., including 16 from the AA1_1 category and 3 from the AA2 category (Figure 4B). Different from species of *Lyophyllaceae*, *Termitomyces* are symbiotic fungi, which depend on fungus-growing termites. Termites use their fecal pellets from plant materials as the growing media to cultivate *Themitomyces* mycelia (Aanen et al., 2007). Termites feed on lignocellulose, remove 74%–99% of the cellulose and 65%–87% of the hemicellulose components, and defecate lignin-rich feces (Brune, 2014). Also the media of *Termitomyces* sp. is mainly lignin-riched feces, the accessibility of cellulose is higher than those of raw lignocellulose materials. Therefore, the lignin-degrading enzymes were lost during the evolution.

In addition to AAs, 253 proteins from GH family were identified in the genome *L. decastes*. There proteins were subclassified into 55 GH families and they were mainly distributed in GH5, GH18, GH3, and GH43 families. Proteins in GH families were cellulose and hemicellulose degradation related enzymes, which suggested that *L. decastes* could use cellulose and hemicellulose as the substrate for mycelial growth.

### Identification of the mating genes and marker development

**Figure 5:**
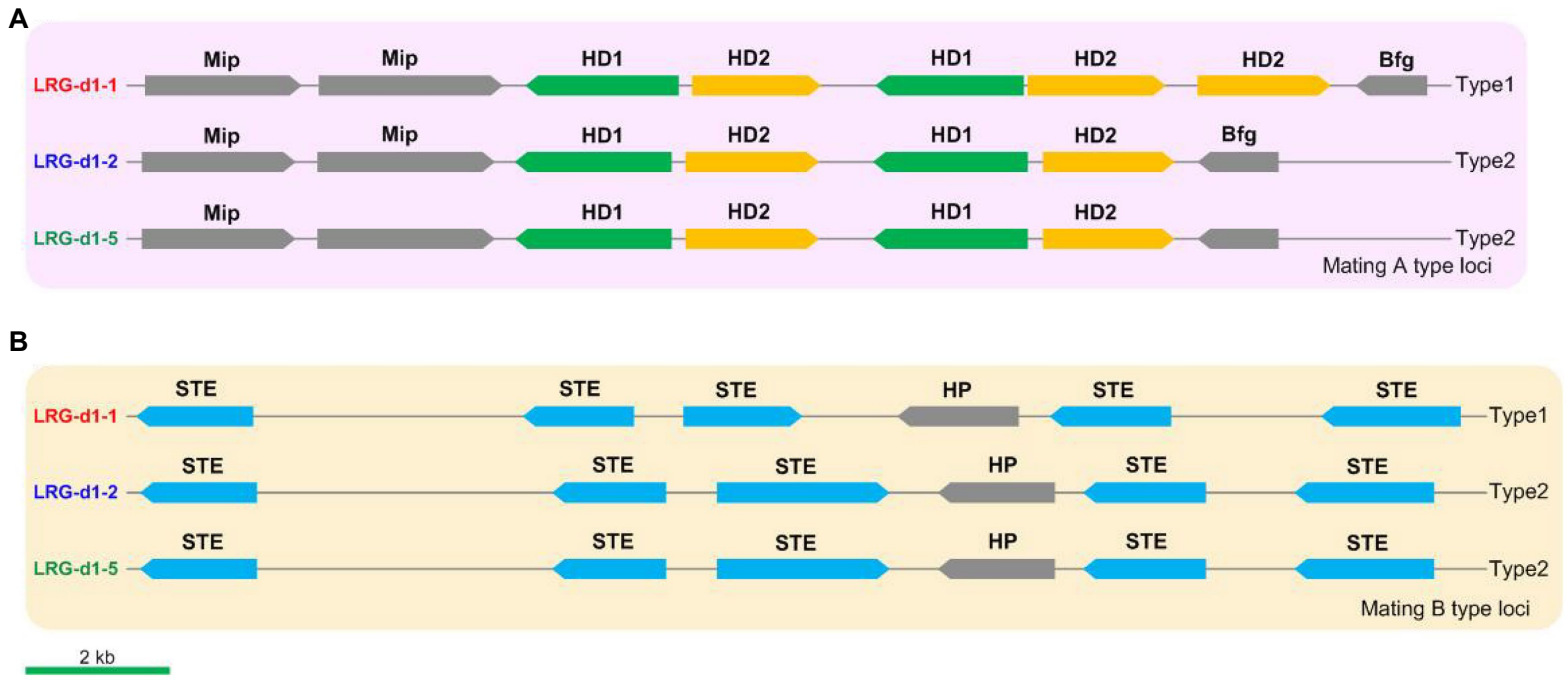
The structures and types of mating loci in *Lyophyllum decastes*. **(A)** The mating A structure of *L. decastes*. **(B)** The mating B locus of *L. decastes*.

The mating loci of *L. decastes* were identified using Blastp tools against mating-related proteins in relative species such as *H. marmoreus* and *P. giganteus*. Five *HD* genes, including 2 *HD1* genes and 3 *HD2* genes, were identified on chr01 in *L. decastes* strain LRG-d1-1 (Figure 5A). The five *HD* genes are grouped into two pairs of homeodomain transcription factor genes, and the organization of these genes is similar to the type1 mating *A* locus in *H. marmoreus* (Wang et al., 2021a). The mating *A* locus of strain LRG-d1-2 and strain LRG-d1-5 has four *HD* genes grouped into two pairs of *HD* transcription factor genes. The organization of these genes is different from all kinds of mating *A* loci in *H. marmoreus* reported by Wang et al. (2021a). The mitochondrial intermediate peptidase (*Mip*) and beta flanking gene (*Bfg*) were located in the two ends of the mating *A* locus of *L. decastes*. The mating *B* locus was identified on chr10 in *L. decastes* strain LRG-d1-1 (Figure 5B). The mating *B* loci of the three *L. decastes* strains all have five *STE*s. The organization of these genes in the three strains is similar, while different from mating *B* loci reported in *H. marmoreus* (Wang et al., 2021).

According to the nucleotide sequence of mating loci in strain LRG-d1-1 and LRG-d1-5, four pairs of primers (Supplementary Table 2) were designed for the PCR-based verification of *L. decastes* mating types. Applicability of the primers was tested in the monokaryotic mycelial isolates raised from the basidiospores of *L. decastes* LRG-d1 and the dikaryotic *L. decastes* strains. Among 8 monokaryotic isolates, all strains yielded two DNA bands either from mating *A* or from mating *B* (Supplementary Figure 3). According to PCR results, the strain LRG-d1-1 is *A1B1* mating type, while the LRG-d1-2 and LRG-d1-5 are *A2B2* mating type, in agreement with the genome information. The three dikaryotic isolates showed four DNA bands from *A1*, *A2*, *B1*, and *B2* mating types (Supplementary Figure 3). The results confirmed that the four primers could precisely distinguish the two different mating types of *L. decastes*.

To further validate the effectiveness of the primers in cross-breeding, mating experiments based on PCR results were performed. The monokaryotic isolates with different *A* mating types and different *B* mating types were placed on one PDA plate at a close distance. After 2 weeks of incubation, mycelial samples from the growing edge on the perpendicular bisector between two inoculation blocks. The mating type of the samples was measured using PCR with the mating type-specific primers and the micromorphology of the samples was checked using a microscope. Our results confirmed that the monokaryotic strains with compatible mating types could form fertile dikaryotic mycelia with clamp connections (Supplementary Figure 3). The results confirmed that *L. decastes* is a tetrapolar heterothallism mating type species. Identification of the exact mating type of a monokaryotic strain can reduce the cross-breeding time. Therefore, the results presented in this study contribute to the *L. decastes* breeding industry through accelerating of the cross-breeding process.

### Secretion of lignocellulose-degrading enzymes by *Lyophyllum decastes*

**Figure 6:**
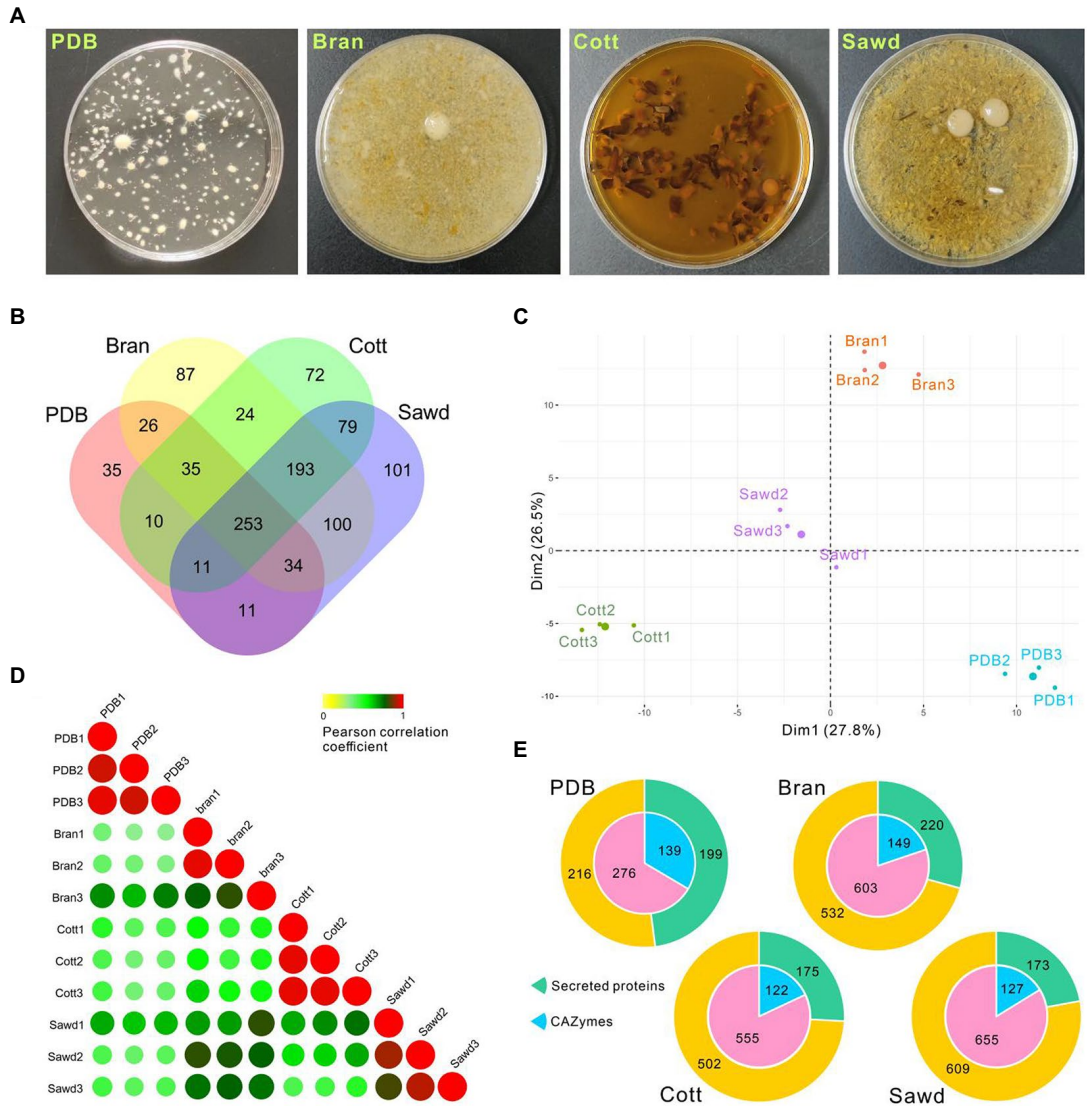
Proteomic analysis of *Lyophyllum decastes* secretome in different media. **(A)** Liquid cultivated *L. decastes* mycelia in different media. **(B)** Venn plot showing the identified proteins in four groups. **(C)** PCA of the first principal components based on common proteins identified from PDB, Bran, Cott, and Sawd groups. **(D)** Pearson correlation coefficients for pair-wise comparisons of proteome data from 12 samples. **(E)** Pie plots represent the identified CAZymes and secreted proteins of each group by proteomic analysis. Proteins that have a signal peptide and no trans-membrane region were recognized as secreted proteins.

As a wood-rotting fungus, farmers normally use lignin-rich sawdust from broad-leaved trees and cotton hull as carbon sources and use bran as the nitrogen source for *L. decastes* fruiting body production. Efficient lignocellulose utilization is one of the key factors to increase mushroom yield. However, the ability of *L. decastes* to degrade lignocellulose is still not clear. To investigate the molecular mechanism underlining lignocellulose degradation by *L. decastes*, the secretome of *L. decastes* in PDB media (PDB group), PDB + bran media (Bran group), PDB + cotton hull media (Cott group), and PDB + Sawdust media (Sawd group) were analyzed (Figure 6A) using label-free proteomic analysis.

A total of 1,071 proteins were identified in 12 samples from *L. decastes* supernatant of 4 groups (three biological replicates for each group; Supplementary Table 6), and 415, 752, 677, and 782 proteins were identified in the PDB, Bran, Cott, and Sawd groups, respectively (Figure 6B). Among identified proteins, only 23.6% (253) proteins were identified in all groups. Interestingly, 18.0% (193) proteins were identified in three lignin-rich groups but not in the PDB group. It is worth noting that the protein number identified in lignin-rich media were much higher than those in the PDB group. However, the number of secreted proteins or CAZymes are similar between different groups (Figure 6E). A similar result was also observed in *P. ostreatus* that straw and poplar revealed a strong and common effect of lignocellulose on the secreted proteins (Fernandez-Fueyo et al., 2016). The result indicated that all three lignin-rich substrates could induce the expression of a more complex secretome than PDB media. PCA results revealed that samples from the same group clustered while samples from different groups were divided into four groups by the first and second components (Figure 6C). Pearson correlation coefficients analysis revealed similar results, that samples from the same group have high correlation values while samples from different groups have lower correlation values (Figure 6D). The results suggested that the protein expression levels induced by different substrates were different despite similar protein numbers identified in the three lignin-rich groups.

Four media used in this study were all derived from PDB media, which contain 20 g/L (111 mM) glucose. High glucose concentration could inhibit cellulose hydrolysis activity and the utilization of hemicellulose (Ohmine et al., 1983; Nijland et al., 2019). Therefore, a high concentration of glucose might inhibit the expression of cellulose and hemicellulose degradation enzymes by so-called feedback inhibition. As expected, the expressions of cellulose and hemicellulose degradation enzymes in *L. decastes* were not significantly induced by the lignin-rich substrates. Twenty-one cellulose degradation enzymes from GH families were identified through proteomic analysis. However, the total abundance in the lignin-rich groups is not induced compared with the PDB group. Xylan hemicelluloses are considered the second most abundant class of polysaccharides after cellulose. Three xylosidases and one xylanase were identified through proteomic analysis (Supplementary Table 7), and three were only identified in lignin-rich groups. Alpha-xylosidase, MDBLdec1_11857, has the highest abundance among the four proteins, and it was identified in all three lignin-rich goups but PDB group. The results indicated that xylan in lignocellulose substrates might induce the expression of xylan degradation enzymes in lignin-rich groups. Even so, the abundance of these enzymes is quite low.

**Figure 7:**
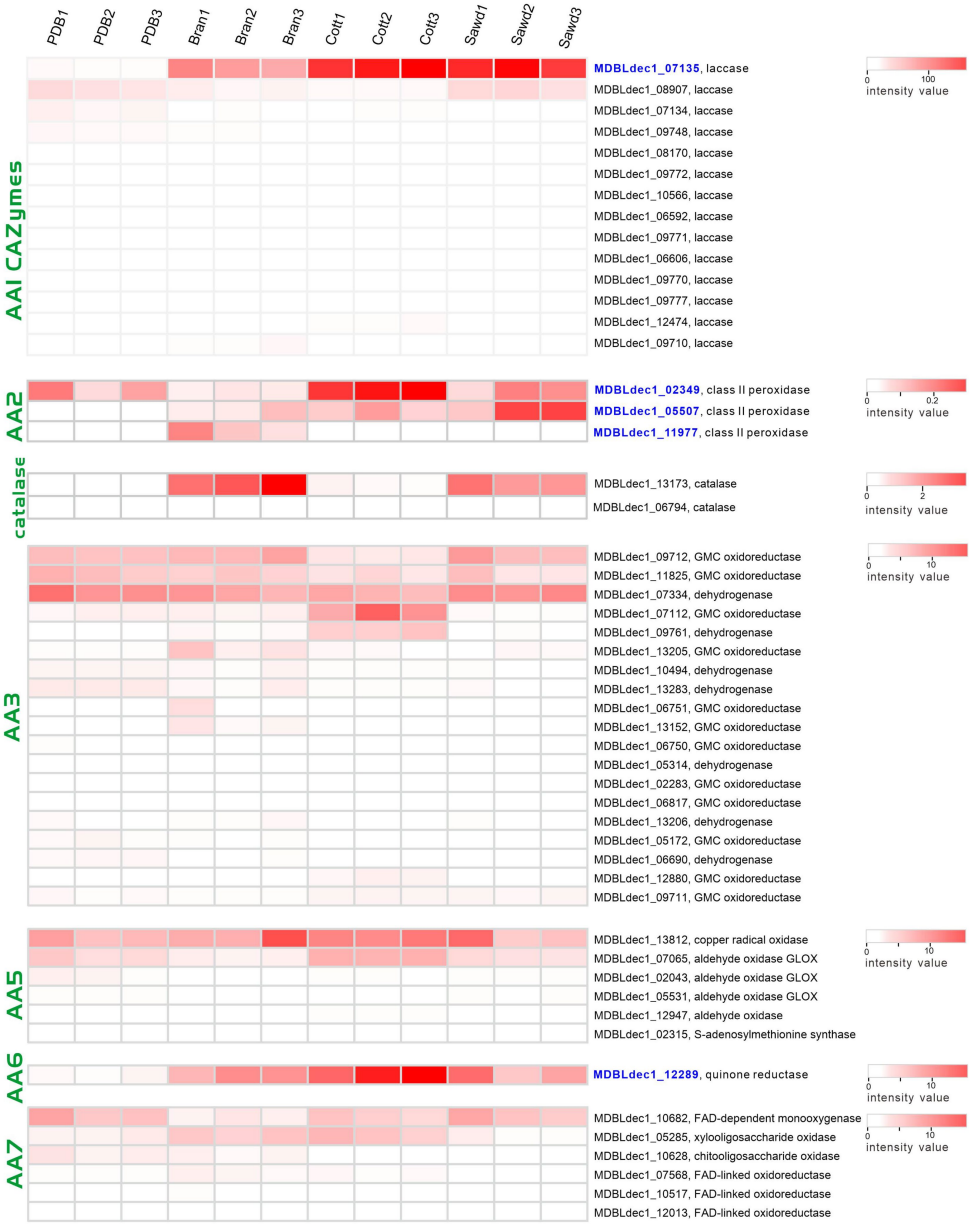
Heatmap of the lignin modification enzymes (LMEs) identified in four groups. The color represents the value of the normalized abundance of each protein.

Laccases (AA1 CAZymes family) and class II peroxidases (AA2 CAZymes family) are two major classes of enzymes involved in the modification of lignin. Three class II peroxidases and 17 laccases were identified from four proteome groups. The abundances of these genes were shown with a heatmap plot in Figure 7. Obviously, the abundances of two laccases (MDBLdec1_07135, MDBLdec1_09727) in the lignin-rich groups are significantly higher than those in the PDB group and much higher than those of other identified laccases. Furthermore, the abundances of these two laccases are among the highest-three abundance proteins in lignin-rich groups (Supplementary Table 7). Similar results have also been observed in other white-rot fungi. Fernández-Fueyo reported that the abundance of laccase increased from 1% of the total protein abundances in non-lignin media to 21% in the lignin-rich media (Fernandez-Fueyo et al., 2016). Therefore, MDBLdec1_07135 and MDBLdec1_09727 are considered to play essential roles in the lignin degradation in *L. decastes*. MDBLdec1_07135 and MDBLdec1_09727 show the highest sequence identity with 66,615 and 123,288 proteins in PC9, respectively. However, the two proteins were not identified in the secretome in *P. ostreatus* PC9 (Fernandez-Fueyo et al., 2016), suggesting different regulation pathways between the two species. Besides, some other laccases, such as MDBLdec1_09748, MDBLdec1_08907, and MDBLdec1_07134, also show relatively high expression abundance in four groups; however, these laccases were not induced in lignin-rich media. Starch usually co-exists with lignocellulose in nature, therefore, the expression of these laccases might be induced by the starch or other polymer in potato extract.

Heatmap of the AA2 family proteins revealed that two peroxidases (MDBLdec1_05507, MDBLdec1_11977) were only expressed in lignin-rich groups. The abundance of MDBLdec1_02349 in the Cott group is significantly higher than that in the PDB group. The results indicated that despite the different structures of the lignocellulose substrates of the three lignin-rich groups the expression patterns of laccases and class II peroxidases in the three groups are similar. The three substrates might produce the same inducer for the induction expression of these enzymes. Peroxidase catalyzing reaction requires H_2_O_2_ as the co-substrate for lignin degradation. White rot fungi secreted many partner enzymes that co-expressed with peroxidases, such as glucose-methanol-choline oxidoreductases from the AA3 CAZymes family, glyoxal oxidases (AA5 family) and oligosaccharide oxidases (AA7 family). In our proteomic analysis, 23, 6, and 7 proteins from AA3, AA5, and AA7 families were identified from four groups (Figure 7), respectively. These enzymes oxidize a variety of carbohydrates, alcohols, and aldehydes derived from lignocellulose. The results indicated that three lignocellulose substrates, sawdust, cotton hull, and bran, were degraded to different products, and the expression of these peroxidase partner enzymes may be induced by corresponding substrates. H_2_O_2_ could not only serve as a co-substrate it could also inhibit the lignin-active peroxidase activity. Therefore, white rot fungi produced the H_2_O_2_-scavenging enzyme, catalase, to regulate the high concentration of H_2_O_2_. Two catalases, MDBLdec1_06794 and MDBLdec1_13173 were identified in proteomic analysis. Interesting, MDBLdec1_13173 abundances in Bran and Sawd groups were significantly higher than that in PDB and Cott groups and MDBLdec1_06794 was only expressed in Bran and Sawd groups (Figure 7). The results indicated that different substrates induced different peroxidase expression patterns, which further influence the expression of partner enzymes such as H_2_O_2_-generating and H_2_O_2_-scavenging partner enzymes. Besides, catalases expression are usually associated with stress response *in vivo*. Therefore, the extracellular expression of catalases might also influenced by the stress response in mycelia.

Laccases and class II peroxidases transform lignin into reactive quinones and phenoxy radicals *in vitro*, which leads to the formation of quinone and (re)polymerized lignin. To efficiently depolymerize lignin, partner enzymes in the white-rot fungi secretome are required to deactivate laccase- and peroxidase-generated quinones and phenol radicals (Monclaro et al., 2022). These enzymes include dehydrogenases from the AA3 CAZymes family and quinone reductases from the AA6 CAZymes family. Here, we identified an AA6 family quinone reductase (MDBLdec1_12289) in proteomic analysis. Compared with the abundance of MDBLdec1_12289 in the PDB group, the abundances in Bran, Cott, and Sawd groups increased by 12.4-fold, 27.7-fold, and 12.4-fold, respectively (Figure 7). It was reported that AA6 quinone reductase could increase the lignin depolymerization by Lip *in vitro* (Majeke et al., 2021). The results suggested that MDBLdec1_12289 played important role in lignin decomposition in *L. decastes*.

Laccases generally oxidize a wide range of substrates, such as lignin; typically transform phenols and aromatic amines into phenoxy radicals. These unstable chemical products commonly start domino reactions to form phenols or quinones (Arregui et al., 2019; Janusz et al., 2020). Quinones can be used by quinone reductases, laccases, and peroxidases in redox cycle reactions to activate oxygen which could further produce hydroxyl radicals. Hydroxyl radicals are among the strongest oxidants in white-rot fungi cultures and can initiate the attack on lignocellulose, which leads to the depolymerization of lignin (Gasser et al., 2012). Cuillen et al. reported the existence of quinone redox cycling in white-rot fungi *Pleurotus eryngii*. Therefore, the results suggested that the two laccases (MDBLdec1_07135 and MDBLdec1_09727) and one quinone reductase (MDBLdec1_12289) play an essential role and have a synergistic effect on lignin decomposition in *L. decastes* LRG-d1 (Guillen et al., 1997). However, further work is needed to characterize the role of laccase and quinone reductases in lignin decomposition.

## Conclusion

In the present study, the high-quality genomes of three mating compatible monokaryotic strains of *L. decastes* were sequenced using Oxford Nanopore and Illumina sequencing platforms. Comparative genomic analysis and phylogenetic analysis revealed that *L. decastes* has a close evolutionary relationship with another industrially cultivated mushroom *H. marmoreus*, and *L. decastes* split from *H. marmoreus* ~ 45.53 Mya ago. The two mating types of *L. decastes* were presented and mating type-specific primers were designed to precisely distinguish different mating types of *L. decastes* by PCR-based method, which help us to accelerate the efficiency of cross-breeding. Based on the integrated genomic and proteomic analysis, lignocellulose degradation potential and secretome in different media were investigated. Laccases and quinone reductase that are significantly up-regulated in lignin-rich media could be the target for further research on lignin degradation and enhance the growth rate in genetic breeding. This study not only provides a representative genome resource for edible mushrooms but also provides useful information for *L. decastes* breeding.

## Data availability statement

Genome sequence data have been deposited in GenBank under accession number CP107701-CP107715 and JAOWSY000000000 for strain LRG-d1-1 and LRG-d1-5, respectively. The whole genome sequence data reported in this paper have been deposited in the Genome Warehouse in National Genomics Data Center (Chen et al., 2021; CNCB-NGDC Members and Partners 2022) under accession number GWHBOVF00000000, GWHBOVC00000000, and GWHBOVD00000000, respectively, that is publicly accessible at https://ngdc.cncb.ac.cn/gwh. The raw sequence data reported in this paper have been deposited in the Genome Sequence Archive in National Genomics Data Center with accession number of GSA: CRA008479, CRA008481, and CRA008488, respectively, that are publicly accessible at https://ngdc.cncb.ac.cn/gsa (CNCB-NGDC Members and Partners 2022). The raw data for the proteomic analysis reported in this paper have been deposited in the OMIX, China National Center for Bioinformation/Beijing Institute of Genomics, Chinese Academy of Sciences (https://ngdc.cncb.ac.cn/omix:accessionno.OMIX002140) (CNCB-NGDC Members and Partners 2022). Genome sequence data is publicly accessible at ftp://www.mushroomlab.cn/.

## Author contributions

LX: formal analysis, methodology, and writing—original draft. WY: data curation and project administration. TQ: methodology and visualization. XG: project administration. HZ and LG: resources. SZ: validation. HC: validation. HaiY: conceptualization and writing—review and editing. HaoY: conceptualization, formal analysis, and writing—review and editing. All authors contributed to the article and approved the submitted version.

## Funding

This research was funded by Key R&D Program of Shandong Province (2021ZDSYS28), Shandong Edible Fungus Agricultural Technology System (SDAIT-07-02), and Breeding and protection of a new variety of Hypsizygus marmoreus (660-2418103).

## Conflict of interest

The authors declare that the research was conducted in the absence of any commercial or financial relationships that could be construed as a potential conflict of interest.

## Publisher’s note

All claims expressed in this article are solely those of the authors and do not necessarily represent those of their affiliated organizations, or those of the publisher, the editors and the reviewers. Any product that may be evaluated in this article, or claim that may be made by its manufacturer, is not guaranteed or endorsed by the publisher.

## Footnotes

^1^ https://github.com/lbcb-sci/racon
