# Supplementary material for: Complete genome sequences and comparative secretomic analysis for the industrially cultivated edible mushroom Lyophyllum decastes reveals insights on evolution and lignocellulose degradation potential
Source: Front Microbiol. 2023 Mar 23;14:1137162. doi: 10.3389/fmicb.2023.1137162 (PMC10078946; doi:10.3389/fmicb.2023.1137162)
Supplement: Supplementary file 2 [file Data_Sheet_1.docx]

**Complete Genome Sequences and Comparative Secretomic Analysis for the Industrially Cultivated Edible Mushroom *Lyophyllum decastes* Reveals Insights on Evolution and Lignocellulose Degradation Potential**

Lili Xu^§1,2^, Wujie Yang^§3^, Tianmei Qiu^1^, Xia Gao^3^, Hongyong Zhang^4^, Shuliang Zhang^4^, Hui Cui^3^, Lizhong Guo^1^, Hailong Yu*^2^, Hao Yu*^1^

^1^Shandong Provincial Key Laboratory of Applied Mycology, School of Life Sciences, Qingdao Agricultural University, 700 Changcheng Road,Chengyang District, Qingdao, Shandong Province, 266109, People'sRepublic of China;

^2^National Engineering Research Center of Edible Fungi, Institute of Edible Fungi, Shanghai Academy of Agricultural Sciences, Shanghai, China;

^3^Shandong Agricultural Technology Extending Station, Jinan, Shandong Province, 250100, PR China;

^4^Dezhou Academy of Agricultural Sciences, Dezhou, Shandong Province, China.

§Lili Xu and Wujie Yang contributed equally to this work.

*Corresponding author:

Hao Yu E-mail: yuhaosunshine@163.com

Hailong Yu E-mail: yuhailong@saas.sh.cn

**Key words:** edible mushroom; *Lyophyllum decastes*; genome; proteome; secretome; CAZymes

Runing Title: *Lyophyllum decastes* Genome and secretome

**Table S1 Repeat sequences in L. decastes genome identified using RepeatMasker.**

|  | number of elements | length occupied | percentage of sequence |
| --- | --- | --- | --- |
| Retroelements | 6822 | 8997636 bp | 18.86% |
| SINEs: | 51 | 9972 bp | 0.02% |
| Penelope | 0 | 0 bp | 0.00% |
| LINEs: | 776 | 1541659 bp | 3.23% |
| CRE/SLACS | 0 | 0 bp | 0.00% |
| L2/CR1/Rex | 0 | 0 bp | 0.00% |
| R1/LOA/Jockey | 31 | 37293 bp | 0.08% |
| R2/R4/NeSL | 0 | 0 bp | 0.00% |
| RTE/Bov-B | 0 | 0 bp | 0.00% |
| L1/CIN4 | 0 | 0 bp | 0.00% |
| LTR elements: | 5995 | 7446005 bp | 15.60% |
| BEL/Pao | 16 | 20280 bp | 0.04% |
| Ty1/Copia | 661 | 604711 bp | 1.27% |
| Gypsy/DIRS1 | 2457 | 5092292 bp | 10.67% |
| Retroviral | 0 | 0 bp | 0.00% |
|  |  |  |  |
| DNA transposons | 589 | 794413 bp | 1.66% |
| hobo-Activator | 0 | 0 bp | 0.00% |
| Tc1-IS630-Pogo | 191 | 139631 bp | 0.29% |
| En-Spm | 0 | 0 bp | 0.00% |
| MuDR-IS905 | 0 | 0 bp | 0.00% |
| PiggyBac | 0 | 0 bp | 0.00% |
| Tourist/Harbinger | 21 | 24977 bp | 0.05% |
| Other (Mirage,P-element, Transib) | 0 | 0 bp | 0.00% |
|  |  |  |  |
| Rolling-circles | 53 | 129067 bp | 0.27% |
|  |  |  |  |
| Unclassified: | 4197 | 1976810 bp | 4.14% |
|  |  |  |  |
| Total interspersed repeats: |  | 11768859 bp | 24.66% |
|  |  |  |  |
| Small RNA: | 13 | 93669 bp | 0.20% |
|  |  |  |  |
| Satellites: | 0 | 0 bp | 0.00% |
| Simple repeats: | 4837 | 209975 bp | 0.44% |
| Low complexity: | 868 | 46541 bp | 0.10% |

**Table S2 Primers used for mating locus identification.**

| Primer name | Sequence (5′–3′) |
| --- | --- |
| matA-I-F | TATAAAACCAGAATCTCCCA |
| matA-I-R | CCGGACTCCTATGACGGTGC |
| matA-II-F | GTTTCCCATCTGGAAAATTG |
| matA-II-R | CAGAAATGCTGACACTTATG |
| matB-I-F | GCAGAACCGACAGCATGGAA |
| matB-I-R | GGATCAAGCCTCAAGTCAAC |
| matB-II-F | GCCTGTCACCGAAGTCACT |
| matB-II-R | CGTTATAATGTCTCAACGC |

**Figure S1**


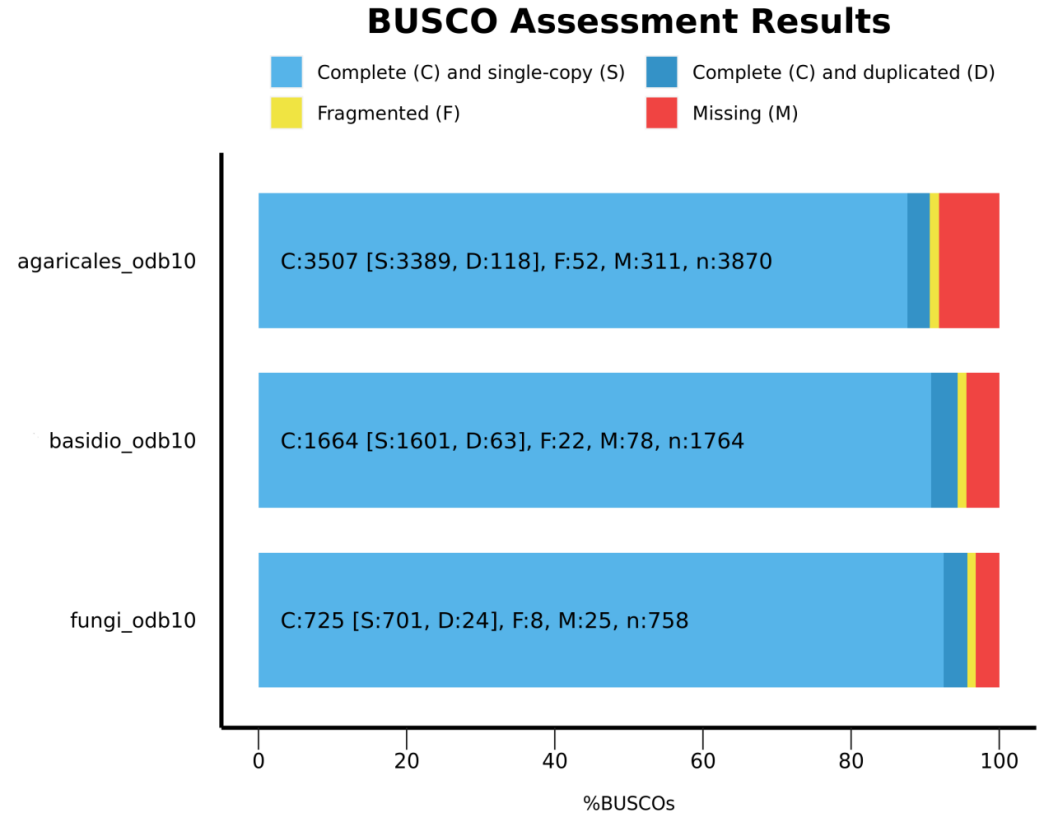


**Figure S1 BUSCO assessment of the protein annotation completeness in *L. decastes* with different BUSCO databases.** The completeness of gene prediction was evaluated using fungi_odb10 (95.7%), basidio_odb10 (94.4%), and agaricales_odb10 (90.6%) databases, respectively.

**Figure S2**


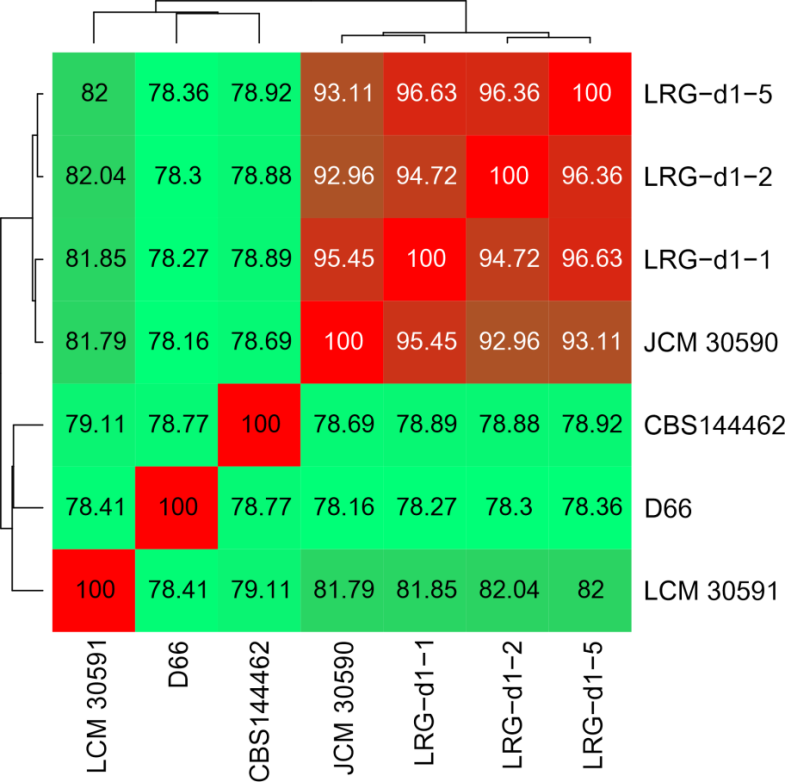


**Figure S2 The average nucleotide identity (ANI) values based on the fastANI algorithm generated matrix for *Lyophyllaceae* genomes.** The clustering was constructed using Euclidean distance matrix.

**Figure S3**


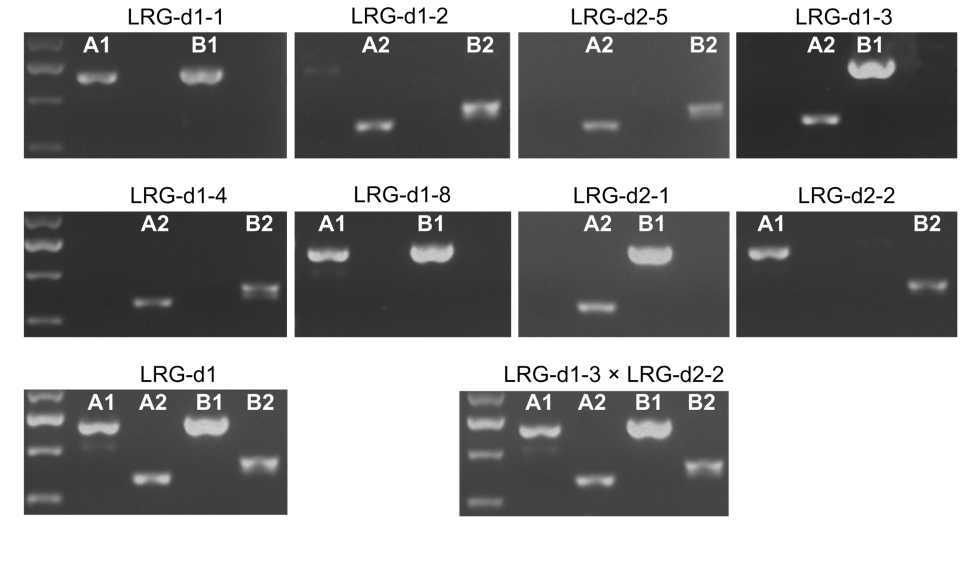


**Figure S3 Measurement of mating types of dikaryotic, monokayotic, and hydride *L. decastes* strains based on mating type specific primers.**
